# Matrix Metalloproteinases MMP-2 and MMP-9, Their Inhibitors TIMP-1 and TIMP-2, Vascular Endothelial Growth Factor and sVEGFR-2 as Predictive Markers of Ischemic Retinopathy in Patients with Systemic Sclerosis—Case Series Report

**DOI:** 10.3390/ijms21228703

**Published:** 2020-11-18

**Authors:** Arleta Waszczykowska, Michał Podgórski, Michał Waszczykowski, Zofia Gerlicz- Kowalczuk, Piotr Jurowski

**Affiliations:** 1 Department of Ophthalmology and Vision Rehabilitation, Medical University of Lodz, Zeromskiego 113, 90-549 Lodz, Poland; piotr.jurowski@umed.lodz.pl; 2 Department of Diagnostic Imaging, Polish Mother’s Memorial Hospital Research Institute, Rzgowska 281/289, 93-338 Lodz, Poland; chilam@tlen.pl; 3 Department of Arthroscopy, Minimally Invasive Surgery and Sports Traumatology, Medical University of Lodz, Zeromskiego 113, 90-549 Lodz, Poland; mwaszczykowski@wp.pl; 4 Department of Dermatology, Pediatric Dermatology and Dermatological Oncology, Medical University of Lodz, Kosciuszki 4, 90-647 Lodz, Poland; zofia.gerlicz-kowalczuk@umed.lodz.pl

**Keywords:** scleroderma, MMP-2, MMP-9, TIMP-1, TIMP-2, VEGF, sVEGFR-2, ischemic retinopathy, angiogenesis

## Abstract

Systemic sclerosis (SSc) is an autoimmune connective tissue disorder associated with multiple organ involvement. The aim of the study was to present two SSc patients who were diagnosed with ischemic retinopathy in both eyes. As a background to our case study, we decided to investigate the imbalance of angiogenesis factors in 25 SSc patients in relation to 25 healthy controls. Assays of matrix metalloproteinases-2 and -9 (MMP-2, MMP-9), tissue inhibitor of metalloproteinases-1 (TIMP-1) and -2 (TIMP-2), vascular endothelial growth factor (VEGF), and soluble VEGF receptor-2 (sVEGFR-2) in blood serum and tears were performed. A significantly increased levels of MMP-9 in serum and tears, (*p* = 0.0375 and *p* < 0.001, respectively) as well as VEGF/sVEGFR-2 ratio in tears (*p* < 0.001) were found in the whole SSc patients group compared with controls, while reduced levels of these parameters in patients with ischemic sclerodermic retinopathy were noted. We also observed decreased level MMP-2 in tears and increased levels of TIMP-2 in blood serum and tears of SSc patients with retinal ischemic changes. MMP-9, MMP-2, TIMP-2, and VEGF/sVEGFR-2 may play a crucial role in ischemic retinal degeneration or retinal reorganization in SSc.

## 1. Introduction

Systemic sclerosis (SSc; scleroderma), is a rare, severe, and chronic connective tissue disease of unknown etiology [1], manifested by cutaneous sclerosis and variable systemic involvement [2].

Abnormal accumulation of extracellular matrix (ECM) components leading to tissue fibrosis, autoimmunity, and endothelial cell damage responsible for tissue hypoxia are the most significant features of the disease [3,4]. Hypoxia is the primary stimulus to modulate the expression of angiogenic and matrix regulatory proteins which are responsible for mediating angiogenesis, cell proliferation, and migration. In the course of SSc, chronic inflammation of microcirculation vessels stimulates lymphocytes to produce metalloproteinases, which play a key role in the degradation of ECM components [2].

Matrix metalloproteinases (MMPs) may interact directly with structural elements of ECM or indirectly, due to their proteolytic release of cytokines and growth factors, including vascular endothelial growth factor (VEGF) [5] One of the principal proteins of the MMP family in the vasculature are the gelatinases (MMP-2 and -9) regulating vascular matrix remodeling [6]. Increased peripheral concentrations of these MMPs have been reported in patients with acute coronary syndromes [7], neuroinflammatory processes [8], and scleroderma [9]. The action of metalloproteinases is controlled at the stage of activation of their progenitor forms; they may be also suppressed by specific inhibitors, tissue inhibitors of MMPs (TIMPs) [3]. TIMP-1 expression is regulated by several proinflammatory cytokines whereas TIMP-2 seems to be independent of cytokine action [10]. Recent studies on the role of metalloproteinases in ophthalmic diseases brought hope for understanding the pathomechanisms of many disorders, as well as their effective prevention and treatment [11,12,13].

We have previously shown the frequency of ocular manifestations in patients with systemic scleroderma [14]. The most serious of them, threatening significant visual impairment in the form of ischemic retinopathy, was observed in 8% of SSc patients [15].

## 2. Aim

The basic aim of the study was to present two SSc patients who were diagnosed with ischemic retinopathy in both eyes. The analysis of these cases has also prompted us to consider factors that may affect the development of retinal ischemic changes in systemic sclerosis course. Thus, the second aim was to assess the role of pro- and antiangiogenic factors in a group of patients with SSc in comparison with healthy control by evaluation the concentration of MMP-2, MMP-9, tissue inhibitor of metalloproteinases-1 (TIMP-1), tissue inhibitor of metalloproteinases-2 (TIMP-2), VEGF, and sVEGFR-2 in blood serum and tears.

## 3. Case Series Presentation

### 3.1. Case 1

Patient 1, a 51-year old woman, was diagnosed with diffuse systemic sclerosis (dSSc) 6 years before the analysis. The main complaints of the patient were related to skin sclerosis of the face, neck, and upper limbs with concomitant finger contractions of both hands for 8 years, severe pain in both wrists that make it difficult to perform daily living activities (Figure S1), and Raynaud phenomenon that persisted for 6 years. The physical examination revealed small ulcerations in the area of the right ankle and digital ulcers (DU) of the 2nd and 3rd fingertips of the right hand, persisting for about 5 years. In the assessment of skin sclerosis, the patient scored 16 points (moderate thickness) on the modified Rodnan skin score (mRss) and active nailfold videocapillaroscopy (NVC) pattern on the Cutolo scale.

The first changes in the face appearance occurred about 8 years earlier and took the form of thinning of the labial red, atrophy of the nasal wings, and skin telangiectasia. Moreover, esophageal atony and lung fibrosis were confirmed. The patient complained about heart rate disorders and exercise dyspnea lasting for about 7 years.

In the subjective ophthalmological examination, the patient reported a feeling of dry and burning eyes and a decrease in visual acuity. One month earlier, the patient had had laser photocoagulation of the left eye retina due to ischemic edema of the macula. The patient was excluded from coexistence of diabetes mellitus.

In the subject examination, best-corrected visual acuity (BCVA) in the right and left eye was 0.3 and 0.5 in the logMar scale, respectively. Intraocular pressure was 16 mmHg in the right eye and 18 mmHg in the left eye.

In the slit lamp examination, the following were observed: thickening of eyelid skin with telangiectasias, superficial conjunctival hyperemia, and features of dry eye disease (DED), reduced tear meniscus, increased amount of residual materials in the tear film, presence of lid parallel conjunctival folds (LIPCOF) which was rated at 3 points, Schirmer I test in the right eye was 5 mm, in the left eye 6 mm; tear film break-up time (TFBUT) value in the right eye: 3 s, in the left eye: 4 s, lysamine green staining on the von Bijsterveld scale in Franck’s modification: 4 points in both eyes and initial posterior subcapsular cataract in both eyes. At the fundus examination, swelling of the posterior pole of the left eye with petechiae along both temporal arcades and constricted and winding blood vessels of the retina of both eyes were observed.

**Figure 1:**
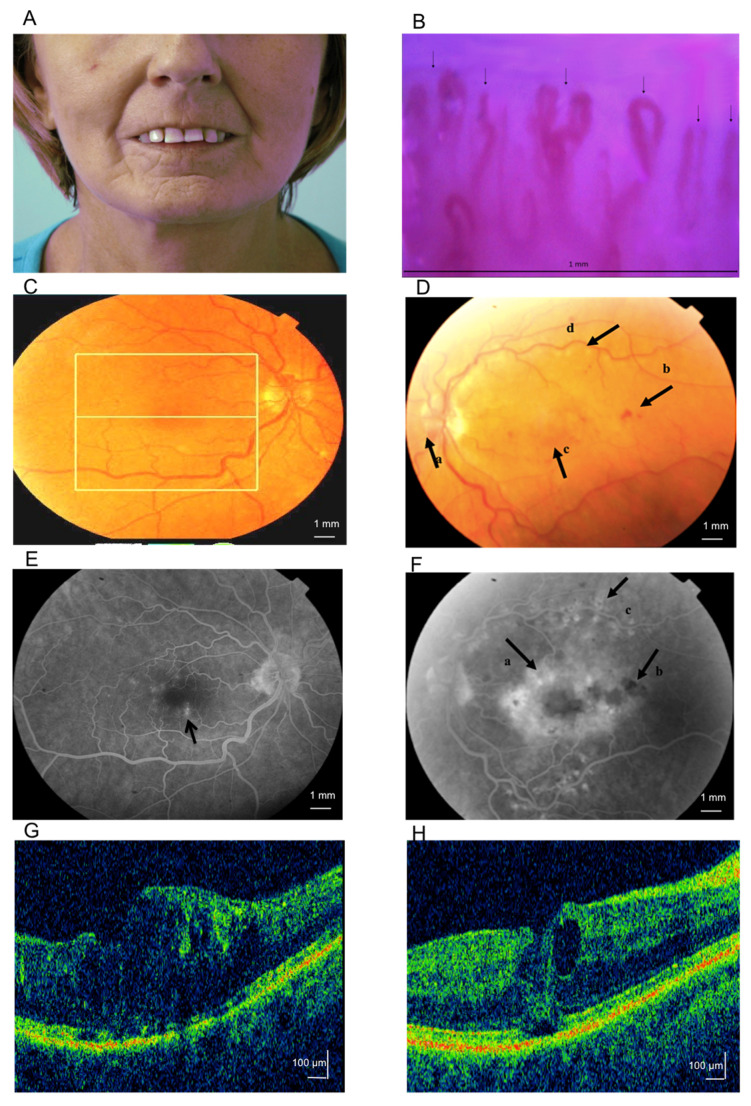
Patient 1, aged 51, diagnosed with systemic sclerosis (SSc) 6 years before the analysis. The image (**A**) shows mask-like facial appearance with characteristic pinched nose, radial furrowing around the mouth, lip thinning and retraction, microstomia, and one single teleangiectasia. (**B**) Nailfold videocapillaroscopy of active SSc pattern in the patient 1 with early desorganization of microvascular architecture; loss of capillaries with lowered density to six capillaries in linear mm; presence of dilated capillary (>20 μm) with abnormal shape and ramification. The capillaries were marked with arrows. Images of right (**C**,**E**,**G**) and left (**D**,**F**,**H**) eye fundus of a patient 1, Data presented as fluorescein angiography (FA) versus optical coherence tomography (OCT). Color photo (**C**) and FA results (**E**) of the right eye fundus: in the recirculation phase, hyperfluorescence foci by a leakage from the microaneurysms can be observed (arrow). Color photo of the left eye fundus (**D**): vasodilation of the optical disc (a), intraretinal hemorrhages (b), macular edema (c), sectional narrowing of retinal vessels (d) may be noted. Results of fluorescein angiography of the left eye (**F**): diffuse macular leakage (a), foci of fluorescence blockade (b), laser photocoagulation scars (c) can be seen. Sclerodermic macular edema in right (**G**) and left eye (**H**) OCT examination.

The optical coherence tomography (OCT) examination result of the right eye perifoveal increased retinal thickness, and extensive, poorly limited increased retinal thickness as a result of extensive collapse of the vascular–retinal barrier of the left eye showed. The fluorescein angiography (FA) analysis confirmed the foveal avascular zone (FAZ) enlargement of the right eye with perifoveal transudate as well as ischemic and extensive macular edema of the left eye. The results and their description are presented in Figure 1 and Figure S1.

Laboratory studies indicated elevated C-reactive protein (CRP) concentration (4.7 mg/dL) and normal fasting glycemia (84 mg/dL). The serological tests showed anti-nuclear antibodies (ANA) anti-topoisomerase I (anty-Scl-70), and anti-Ro52 antibodies presence.

The patient was repeatedly hospitalized in the Department of Dermatology due to episodes of exacerbation of the disease. Until the examination, the patient was treated with Mycophenolate mofetil (treatment included 3 years earlier by a pulmonologist but due to poor tolerance—pain, dizziness, malaise, in morphology: pancytopenia—it was decided to stop the cytostatic therapy). For 5 years the patient was treated with cyclophosphamide (Endoxan) and low doses of steroids (Metypred 8 mg), which were discontinued 2 weeks before the planned ophthalmological examination. In addition, the patient used vitamin E, Omeprazolum (Bioprazole), pentoxyfilline (Polfilin), mucolytic agent (Acetylcysteine), Benzodiazepine (Oxazepam), and Dextran infusions every 6 months during hospitalization.

### 3.2. Case 2

Patient 2, a 64-year-old woman, diagnosed with limited systemic sclerosis (lSSc) 10 years before the analysis.

**Figure 2:**
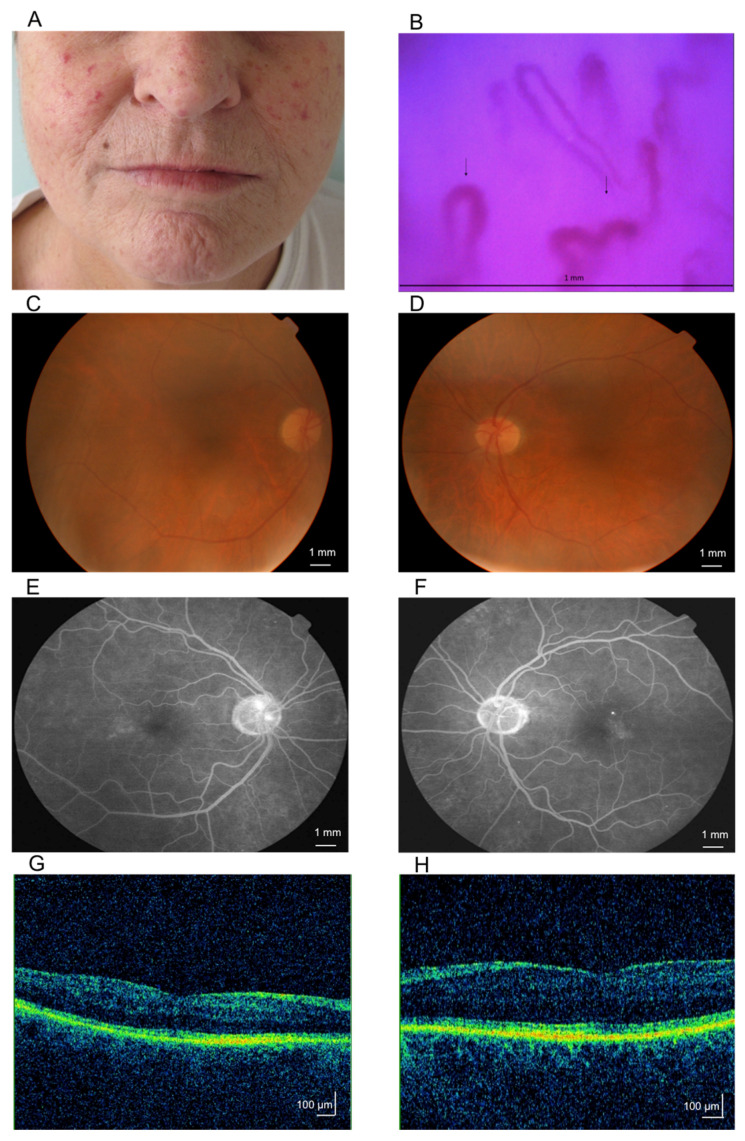
Patient 2, aged 64, diagnosed with systemic sclerosis (SSc) 10 years before the analysis. Photograph (**A**) shows a characteristic facial appearance for scleroderma: atrophy of the nasal wings, radial grooves around the mouth, thinning of the labial red and numerous telangiectasias. Nailfold videocapillaroscopy of late SSc pattern in the patient 2 (**B**) with characteristic disorganization of microvascular architecture; loss of capillaries with lowered density to two capillaries in linear mm; presence of dilated capillary (> 20 μm) with abnormal shape and ramification. The capillaries were marked with arrows. Images of right (**C**,**E**,**G**) and left (**D**,**F**,**H**) eye fundus of a patient 2, aged 64, diagnosed with systemic sclerosis 10 years before the analysis. Data presented as fluorescein angiography (FA) versus optical coherence tomography (OCT). Fluorescein angiography in the recirculation phase showed multiple areas of delayed choroidal filling with hyperfluorescence corresponding to the areas of microaneurysms leakage (**E**,**F**).

The first skin pathological changes had the character of the hands (puffy hands) and face swelling and hardening and appeared about 11 years earlier. Then, the joint pain and fingers contractures of both hands gradually intensified, atrophy of the hand and face skin, difficulties in mouth opening. Raynaud phenomenon has been positive for 10 years (Figure 2). Despite the lack of complaints about swallowing difficulties, the esophageal passage was documented in scintigraphy.

The patient’s main complaints concerned dryness of nasal and oral mucous membranes and conjunctivitis, pain with disturbed sensation of fingertips of the both hands in the Raynaud phenomenon course and worsening of exercise tolerance.

The patient was diagnosed with interstitial pulmonary disease, moderate activity of the scleroderma disease (mRss score 16) and an ulceration was reported on the distal phalanx of 3rd finger of the left hand (Figure S1). Naifold microcirculation was evaluated on the late pattern according to the Cutolo scale.

In ophthalmologic history, the patient reported a dry and burning eyes feeling and a decreased of visual acuity. So far untreated ophthalmologically, the last ophthalmic examination took place about 15 years ago.

In the subjective evaluation, BCVA was 0.2 and 0.4 in logMar scale in the right and left eye, respectively. IOP was 18 mmHg in both eyes.

In slit lamp examination the following was observed: thickening of the skin of the eyelids, varicose veins dilatation of subconjunctival and superficial vessels, and symptoms of DED syndrome (reduced tear meniskus; LIPCOF was assessed at 2 points; Schirmer I test in both eyes was 8 mm; TFBUT value in the right eye: 5 s, in the left eye: 4 s; lysamine green staining on the von Bijsterveld scale in Franck’s modification score in the right eye: 3 and left eye: 4). Moreover, the patient had a nuclear cataract of both eyes. Ophthalmoscopy revealed the presence of narrowing, winding retinal vessels, and the thinning of choriocapillaries with the large choroidal vessels visible.

The OCT examination showed increased retinal thickness of the retina of the left eye with perifoveal edema location. The FA examination revealed, in both eyes, ischemic macular edema with a FAZ enlargement and with perifoveal leakage foci. The examination results and their description are presented in Figure 2 and Figure S1.

Laboratory investigations showed elevated CRP concentration (1.7 mg/dL) and normal fasting glycemia values of 81 mg/dL. Serological tests showed the presence of ANA, anti-Scl-70, and anti-Ro52 antibodies.

Until the examination, the patient was taking penthoxyphilin (Trental) and vitamin E.

## 4. Comparison of Patients with SSc with Healthy Control

In patients with SSc the mean value of Rodnan scale was 14.1 points (median 11 points, range 4–35 points). Eighteen patients had mild symptoms, five moderate, and four severe. The mean result of Cutolo scale was median 3 points (range 2–3 points).

Patients with SSc had significantly increased MMP-9 levels in serum and tears compared with the controls. Controls had significantly higher concentration of VEGF and sVEGFR-2 in tears, but VEGF/sVEGFR-2 tears index was higher in SSc patients. In case of coefficients, only MMP-2 serum/TIMP-1 serum and MMP-2 serum/TIMP-2 serum, as well as VEGF/sVEGFR-2 serum did not differ significantly between groups (Table 1 and Table 2). Table 1 presents the exact results of laboratory tests for two SSc patients with ischemic maculopathy (SSc patients 1 and 2) and for both tested groups. Table 2 shows the calculations for coefficients of parameters. In the Table S1 we included the calculation of TIMP, MMP, VEGF, and sVEGFR-2 molecular weights for stoichiometric analysis.

**Figure 3:**
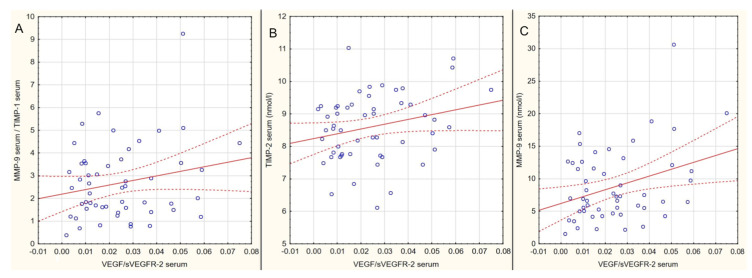
Analysis of all MMPs, TIMPs, VEGF, and sVEGFR-2 tears and blood serum samples from SSc patients and controls. Significant positive correlations were detected between levels of MMP-9/TIMP-1 and VEGF/sVEGFR-2 serum (**A**), TIMP-2 and VEGF/sVEGFR-2 serum (**B**), and MMP-9 and VEGF/sVEGFR-2 serum (**C**).

We observed also significant (although weak) correlations between analyzed parameters that are presented in Table 3 and in Figure 3. Only clinically important parameters were tested for correlations.

### Fundus Fluorescein Angiography of SSc Patients

The detailed characteristics of the eye fundus fluorescein angiography in patients with SSc were presented previously [15]. Ischemic retinopathy was observed in two patients (Figure 1 and Figure 2), one with lSSc and the other with dSSc. Although it is not possible to draw statistically significant conclusions at this stage, it is worth mentioning a few facts. Both patients were in the mean age of the group (51 and 64 years vs. 52.9 ±11.2 years in remaining group) but their Reynaud symptoms did not last as long (6 and 10 years vs. 11.1 ± 5.9 years in remaining group). They had very low concentration of tears MMP-9 (7 and 11 ng/mL) while the median was 19 ng/mL for the remaining SSc patients and 8 ng/mL for controls. Consequently, MMP-9 tears/TIMP-1 tears and MMP-9 tears/TIMP-2 tears coefficients were also much lower. Serum MMP-9 and tears MMP-2 levels were about 2-fold lower than in other patients with SSc. TIMP-2 level in serum and tears was higher compared to the rest of the SSc patients and controls. The detailed characteristics of tested cytokines in blood serum and tears in two patients with ischemic retinopathy compared to the whole group of SSc patients are presented in Table 1 and Table 2.

## 5. Discussion

To the best of our knowledge, ischemic macular edema in SSc patients was not previously described by other authors. Our patients with scleroderma ischemic retinopathy had two different forms of scleroderma, lSSc and dSSc, although both presented moderate activity of the disease. Interestingly, patient 2 with smaller ischemic retinal changes showed more advanced changes in NVC. As a background to our case study, we decided to investigate the imbalance of angiogenesis factors in tears and serum in patients with scleroderma with ischemic maculopathy and no ischemic retinal changes in relation to the control group. Moreover, the analysis of the levels of MMP-2, MMP-9, TIMP-1, TIMP-2, VEGF, and sVEGFR-2 for the evaluation of ischemic retinal microangiopathy and retinal hypoxia were not previously performed.

The involvement of retinal vessels in the course of SSc was previously described only in a few studies and most often took a form of retinal vessel occlusion. This complication occurred at different stages of scleroderma development, sometimes even preceding its diagnosis. The histopathological studies of central retinal artery occlusion in the course of scleroderma showed severe retinal ischemic atrophy, concentric narrowing, and fibrosis of small retinal vessels [16,17,18].

In this study we analyzed total concentration of MMP-2, MMP-9, TIMP-1, TIMP-2, VEGF, and sVEGFR-2 in blood serum and tear fluid in SSc patients. We also attempted to determine associations between the plasma and tear cytokine levels as potential prognostic factors for ischemic retinopathy occurrence in scleroderma course.

The results of the authors’ research indicate that overproduction and accumulation of extracellular matrix components occur in systemic scleroderma. These include type I, III, V, VI collagen and fibronectin, the components of the vascular basement membrane, which explains the stenosis and subsequent closure of the vascular lumen in scleroderma patients. It is believed that the fibrosis process in the course of scleroderma may also be affected by increased synthesis of matrix components or their insufficient degradation due to disorders in the MMP/TIMP system [19,20,21,22]. Further, the closure of capillary lumen observed in the course of systemic scleroderma may lead to hypoxia of tissues. Distler et al. have proven that tissue hypoxia, either short-term or chronic, stimulates fibroblasts of healthy individuals and SSc patients to intensify the expression of many genes and proteins responsible for starting the metabolic pathway of the matrix, which further enhances fibrosis processes [23].

Other reports on the involvement of metalloproteinases in the processes of angiogenesis in the eye are not clear. Some of them suggest that increased levels of MMP-9 have a stimulating effect on the formation of new blood vessels in cornea [24] while others indicate that low collagenase activity and increased collagen production may be an early and sensitive indicator of a high risk of proliferative retinopathy [25].

MMPs, specifically MMP-2 and -9, are one of the most important regulators of angiogenesis with a general tendency to stimulate, and MMPs inhibitors reverse these effects. Degradation of basement membranes and remodeling of the extracellular matrix by MMPs allows endothelial cells to migrate and invade the surrounding tissue during angiogenesis. Moreover, it is believed that gelatinases contribute to angiogenesis through VEGF splitting, which causes irregular blood vessel sprouting and growth. MMP-9 may also control VEGF release from the ECM, and vice versa, VEGF can induce matrix MMP-9 activities and focal angiogenesis [26]. On the other hand, MMPs may exert antiangiogenic effects through the generation of endogenous angiogenesis inhibitors by proteolytic cleavage of certain collagen chains and plasminogen. Both MMP-2 and MMP-9 are interfering with angiogenesis by producing angiostatin, a potent angiogenesis antagonist [27,28,29]. Matrix metalloproteinases, specifically MMP-2 and MMP-9, have been shown to be associated with venous stenosis formation in stenotic samples from animal models of hemodialysis vascular access failure and in ischemic retinas of rats [30,31].

In our study we observed significantly higher levels of MMP-9 and MMP-9/TIMP-1, -2 both in serum and in tears of SSc patients. The predominance of these parameters in patients from the study group was more pronounced in tears compared to blood serum. We did not find a correlation between MMP-2 and any of the investigated clinical parameters in blood serum of SSc patients. In tears, however, we observed significantly higher levels of MMP-2/TIMP-1, -2 which indicates an imbalance in the MMP/TIMP system towards an increased breakdown of matrix proteins in the visual organ during systemic scleroderma.

In both SSc patients with ischemic retinopathy, we observed lower levels of MMP-9 in serum and tears with higher levels of TIMP-2 in serum and tears compared with the whole study and control group. We also observed lower levels of blood serum and tears MMP-9/TIMP-1, -2, lower levels of MMP-2 and MMP-2/TIMP-1, -2 in tears, and significantly lower values of VEGF/sVEGFR-2 in serum compared with the whole group of patients with SSc. This outcome may explain excessive accumulation of matrix proteins, increased fibrosis, and consequently the closure of capillary lumen in retinal microcirculation, without features of neovascularization in these patients. In our previous reports on the role of VEGF and its inhibitor soluble VEGF receptor-2 (sVEGFR-2), we have also shown that higher concentrations of proangiogenic factors may be found in the tears of patients with scleroderma compared with patients’ blood serum and control group [15]. Some previous studies also indicate the major role of VEGF in angiogenesis in systemic sclerosis [32,33,34]. VEGF is involved in many different pathways of angiogenetic processes: initial vasodilation, remodeling of the perivascular matrix, and stimulation of proliferation of endothelial cells [35].

A significant role of TIMPs in angiogenesis has been proven in several reports. TIMP-1 and -2 can inhibit angiogenic responses by inhibiting metalloproteinases. TIMP-2 can inhibit the VEGF-A in a mechanism independent of metalloproteinases also. These effects require *α* 3*β* 1 integrin-mediated binding of TIMP-2 to endothelial cells [36,37]. It has been shown that TIMP-2 can function in the absence of MMPs to maintain cellular differentiation and tissue homoeostasis [38] to suppress cellular responses to transient or minor fluctuations in angiogenic growth factors. Experimental study results on the angiogenesis model demonstrated the proangiogenic activity of TIMP-1 [39]. Significant positive correlations between TIMP-1, MMP-9, and VEGF vitreous fluid levels in proliferative diabetic retinopathy were clinically confirmed [40,41].

In our study, we observed positive correlations between proangiogenic factors: VEGF and MMP-9, VEGF/sVEGFR-2 ratio and MMP-9, as well as VEGF/sVEGFR-2 ratio and TIMP-1 in blood serum. Interestingly, despite the fact that the levels of proangiogenic molecules were significantly higher in tears of SSc patients, positive correlations between antiangiogenic factors occurred only in case of tear sVEGFR-2 and TIMP-2. As tear sampling is a fast and easy standard procedure for ophthalmological examination, evaluation of tear film could be a potentially powerful tool for systemic sclerosis and would provide a noninvasive method for evaluating molecular events associated with the risk of ischemia.

It should be emphasized that our research at this stage contains some limitations. First, since ischemic retinopathy in the scleroderma course is a very rare complication, our sample size was small, and we could not perform a statistical evaluation. Further, the production of MMPs, TIMPs, and VEGF is also induced and regulated by many other factors, including proinflammatory cytokines and growth factors, this aspect in the case of scleroderma requires further studies.

## 6. Materials and Methods

### 6.1. Study Design

For the purposes of our study, in addition to SSc patients with ischemic maculopathy (Patients 1 and 2), we also established a group of patients with systemic sclerosis, which also included Patients 1 and 2, and a control group of healthy individuals.

### 6.2. Participant Selection

Patients 1 and 2 were described previously (see Section 3)

The group of patients with systemic sclerosis consisted of 25 patients (22 females aged 55.7 ± 10.8 years and three males aged 54.9 ± 9.5 years): 17 with limited systemic sclerosis (16 women and one man, aged 38–77 years) and eight with diffuse systemic sclerosis (six women and two men, aged 35–70 years). As no statistically significant differences in the duration Raynaud’s phenomenon occurrence in both clinical forms of sclerosis were found (mean 11.1 ± 5.9 years; though that period in lSSc patients and dSSc patients was mean 14.2 ± 9.4 years; mean: 8.7 ± 5.1 years), all the patients were included into perfusion analysis as one group, without division into clinical forms. All patients fulfilled SSc criteria according to ACR and EULAR [42,43].

The control group consisted of 25 healthy age- and gender-matched individuals (20 females aged 55.7 ± 10.8 years and five males aged 54.9 ± 9.5 years). Exclusion criteria for the control group were chronic general or ophthalmologic diseases.

Exclusion criteria for all study participants were as follows: active infections of the anterior segment of the eye, ocular surgery in the previous six months, glaucoma, uveitis, allergies, thyroid eye disease, diabetes mellitus, chronic use of topical medications, contact lens usage.

Two weeks before the study, the use of artificial tear preparations in all patients group and immunosuppressive drugs in the SSc patients group were discontinued.

This study was conducted in accordance with the Declaration of Helsinki and was approved by the Ethics Committee of the Medical University of Lodz Poland (No RNN/332/06/KB from 29 June 2006). Informed consent was obtained from each patient.

### 6.3. Examination and Image Acquisition

A thorough history of eye diseases was collected from all study participants.

All SSc patients underwent a full history and physical examination. Relevant laboratory and radiological examinations were performed. The activity indices and severity of systemic sclerosis was assessed using modified Rodnan skin score (mRss) [44]. Skin thickening was assessed by palpation of the skin in 17 areas of the body (fingers, hands, forearms, arms, feet, legs and thighs, face, chest and abdomen) using a 0–3 scale, where 0 = normal, 1 = mild thickness, 2 = moderate thickness, and 3 = severe thickness. Total skin score can range from 0 (no thickening) to 51 (severe thickening in all 17 areas).

Patients 1 and 2 underwent a complete ophthalmic examination that included LogMAR best-corrected distance visual acuity (BCVA) measurement with an ETDRS chart (LCD Frey CP-400, Frey Sp.J., Piaseczno, Poland), slit-lamp biomicroscopy with anterior segment, and fundus examination and tonometry (SL-D2 Topcon Inc., Paramus, NJ, USA). The tear film was analyzed using Schirmer filter paper single-use strips (Whatman paper by Clement Clarke) as well as fluorescein and lysamine green staining. Schirmer I test without anesthesia was performed. The wetting strips counted <10 mm per 5 min were defined positive, while ≤5 mm per 5 min were defined strong positive. Tear film break-up time test was performed after fluorescein installation into conjunctival sac. TFBUT was positive below 5 s. The results of lysamine green staining were interpreted according to the von Bijsterveld classification, modified by Franck. The result of staining from three areas of the eye surface > 3.5 points was determined as incorrect [45]. LIPCOF evaluation was performed, without fluorescein, in the area tangential to the temporal and nasal limbus, on the bulbar conjunctiva above the lower lid, using a slit-lamp microscope and ×25 magnification. LIPCOF grade was classified using the Pult LIPCOF grading scale (folds count: 0, no permanent, lid-parallel conjunctival fold; 1, one fold; 2, two folds, 3, three or more folds) [46].

Retinal blood circulation was investigated with fluorescein angiography (FA) in the SSc patients only. The investigation was performed with a Topcon TRC 50 EX fundus camera, an image-net system (Topcon Tokyo Optical Co. Ltd., Tokyo, Japan) after pupil dilatation.

The optical coherence tomography (OCT) was performed in the SSc patients group using a Cirrus high-definition OCT device (5000: Carl Zeiss Meditec Inc., Dublin, CA, USA) or Topcon 3D Optical Coherence Tomograph 1000 (MARK II, version 3.51, Topcon Inc., Paramus, NJ, USA) after mydriasis.

Nailfold microcirculation in the SSc patients group was assessed with the use of the optical microscope OPTEK MC-980 (F.H. Prymus, Ziębice, Poland). After a period of acclimatization to room temperature (22–25 °C) lasting at least 15 min, the nailfold (distal row) of the second, third, fourth, and fifth finger was examined in each patient. Capillaroscopic images are classified as 1—“early”, 2—“active” or 3—“late” SSc patterns, or 0—“normal and specific changes” according to the Cutolo classification [47].

### 6.4. Serum and Tear Samples

Tear and venous blood samples of all participants were taken at a constant time of day between 8 am and 10 am. The collection of tears in patients with systemic sclerosis was carried out during several sessions for consecutive days (mean seven sessions; range 5–13 sessions).

A disposable 5 μL glass microcapillary pipette (Microcaps; Drummonda Scientfic Co., Broomall, PA, USA) was atraumatically placed just inside the lateral cantal margin to collect tears from the inferior fornix when visualized under an ophthalmic microscope. Tear samples were taken without stimulation and without the anesthetic drops administration.

The collected tear fluid was transferred into a 0.5 mL tube (Eppendorf, Fremont, CA, USA) and centrifuged at 6000 rpm, for 5 min at 4 °C, then stored at −80 °C until used.

The blood samples were immediately placed into individual Eppendorf tubes (Eppendorf, Fremont, CA, USA) and centrifuged at 6000 rpm, for 5 min at 4 °C and were stored at −80 °C before use for the assay.

### 6.5. Measurement of Total Blood Serum and Tears MMP-2, MMP-9, TIMP-1, and TIMP-2 Concentration

The total protein concentrations of MMP-2, MMP-9, TIMP-1, and TIMP-2 (ng/mL) were quantified in tears and blood serum of SSc patients and controls, using an enzyme-linked immunosorbent assay (ELISA) kit (Human: MMP-2, MMP-9, TIMP-1, TIMP-2, Quantikene; R&D Systems, Inc., Minneapolis, MN, USA), performed according to manufacturer’s instructions. In MMP-2 and MMP-9 tests the total concentration (proenzymes and activated forms) was determined.

### 6.6. Measurement of Total Blood Serum and Tears VEGF and sVEGFR-2 Concentration

The total protein concentrations of VEGF and sVEGFR-2 (pg/mL) were identified in tears and blood serum of SSc patients and controls using ELISA assays (Human VEGF, Quantikene; R&D Systems, Inc., Minneapolis, MN, USA; Human sVEGFR-2/KDR/Flk-1, Quantikene; R&D Systems, Inc.) following the manufacturer’s protocol.

### 6.7. Statistical Analysis

In statistical analysis, continuous variables are presented as the mean ± standard deviation (SD) unless stated otherwise. Normality of continuous data distribution was assessed with the Shapiro–Wilk test. Differences between continuous variables were evaluated by means of the Mann–Whitney test if two groups were compared or the Kruskal–Wallis ANOVA by ranks with post-hoc test if three groups were evaluated. Statistical analysis was performed using Statistica for Windows (version 13.0, StatSoft, Cracow, Poland). A *p*-value of ≤ 0.05 was considered significant.

## 7. Conclusions

In our study, significantly increased levels of MMP-9 in serum and tears, as well as MMP-2 and VEGF/sVEGFR-2 ratio in tears were found in SSc patients, while reduced levels of these parameters in patients with ischemic sclerodermic retinopathy were noted. Further, an increased level of TIMP-2 in blood serum and tears was observed in these analyzed cases. Based on these results, we speculate that MMP-9, MMP-2, TIMP-2, and VEGF/sVEGFR-2 may play an important role in ischemic retinal degeneration or retinal reorganization in systemic sclerosis.

## Figures and Tables

**Table 1:** Results of laboratory parameters for two patients with ischemic retinopathy (SSc 1 and 2) and comparison of parameters between all SSc patients and controls.

Parameters		SSc Patient 1	SSc Patient 2	Scleroderma Whole Group (Mean (SD))	Control (Mean (SD))	*p*-Value Control vs. Scleroderma Whole Group
MMP-2 (ng/mL)	Serum	246.4	247.2	230.22 (46.401)	249.993 (106.990)	0.5365
	Tears	1.4	1.6	3.03 (6.234)	0.497 (1.204)	0.7048
MMP-9 (ng/mL)	Serum	423	242	839.00 (514.564)	584.600 (405.019)	0.0375 *
	Tears	7	11	244.04 (673.510)	10.633 (10.142)	<0.001 *
TIMP-1 (ng/mL)	Serum	302	229	269.83 (92.782)	245.367 (49.591)	0.1611
	Tears	255	162	234.35 (82.893)	248.700 (43.635)	0.1294
TIMP-2 (ng/mL)	Serum	213	196	188.71 (26.420)	188.700 (25.411)	0.5833
	Tears	212	176	137.73 (42.673)	146.467 (25.313)	0.2624
VEGF (pg/mL)	Serum	392.4	280.3	346.27 (399.88)	197.737 (155.04)	0.4705
	Tears	593.9	41.1	275.30 (552.39)	352.135 (135.25)	<0.001 *
sVEGFR-2 (pg/mL)	Serum	10,445	11,035	10,184.00 (2507.77)	9110.125 (2858.61)	0.0970
	Tears	138.5	39.5	87.25 (69.92)	8274.653 (11,033.51)	<0.001 *

*—significant difference according to the Mann–Whitney test; Abbreviations: SSc—systemic sclerosis; SSc patient 1 and 2—SSc patients with ischemic maculopathy; MMP-2—matrix metalloproteinase-2; MMP-9—matrix metalloproteinase-9; TIMP-1—tissue inhibitor of metalloproteinases-1; TIMP-2—tissue inhibitor of metalloproteinases-2; VEGF—vascular endothelial growth factor; sVEGFR-2—soluble form of vascular endothelial growth factor receptor 2; SD—standard deviation.

**Table 2:** Coefficients of laboratory parameters for two patients with ischaemic retinopathy (SSc 1 and 2) and comparison of coefficients between all SSc patients and controls.

Parameters	SSc Patient 1	SSc Patient 2	Control [Mean (SD)]	Scleroderma Whole Group [Mean (SD)]	*p*-Value Control vs. Scleroderma Whole Group
MMP-2 serum/TIMP-1 serum	0.8116	1.0795	0.920 (0.307)	1.038 (0.409)	0.2451
MMP-2 serum/TIMP-2 serum	1.1568	1.2612	1.232 (0.261)	1.354 (0.675)	0.8896
MMP-2 tears/TIMP-1 tears	0.0055	0.0099	0.016 (0.034)	0.002 (0.006)	0.0452 *
MMP-2 tears/TIMP-2 tears	0.0066	0.0211	0.023 (0.045)	0.004 (0.012)	0.0336 *
MMP-9 serum/TIMP-1 serum	1.4001	1.8515	3.326 (1.973)	2.246 (1.249)	0.0252 *
MMP-9 serum/TIMP-2 serum	1.9859	2.1633	4.664 (2.795)	2.890 (1.593)	0.0201 *
MMP-9 tears/TIMP-1 tears	0.0275	0.0679	1.397 (4.034)	0.049 (0.056)	0.0010 *
MMP-9 tears/TIMP-2 tears	0.0330	0.1447	1.642 (4.323)	0.082 (0.096)	0.0006 *
VEGF/sVEGFR-2 serum	0.0375	0.0254	0.032 (0.030)	0.022 (0.016)	0.1874
VEGF/sVEGFR-2 tears	4.2881	1.0405	3.027 (3.731)	5.285 (7.559)	0.0250 *

*—significant difference according to the Mann–Whitney test; Abbreviations: SSc—systemic sclerosis; SSc patient 1 and 2—SSc patients with ischemic maculopathy; MMP-2—matrix metalloproteinase-2; MMP-9—matrix metalloproteinase-9; TIMP-1—tissue inhibitor of metalloproteinases-1; TIMP-2—tissue inhibitor of metalloproteinases-2; VEGF—vascular endothelial growth factor; sVEGFR-2—soluble form of vascular endothelial growth factor receptor 2; SD—standard deviation.

**Table 3:** Correlations between analyzed parameters in tears and blood serum samples from SSc patients and controls.

Parameters	TIMP-1 Serum	TIMP-2 Tears	MMP-9 Serum	MMP-9 Serum/TIMP-1 Serum
sVEGFR-2 tears	R = 0.2018 p= 0.1555	R = 0.3585 p = 0.0106 ^†^	R = −0.1274 p = 0.3730	R = −0.1288 p = 0.3724
VEGF serum	R = 0.2152 p = 0.1181	R = 0.2413 p = 0.0788	R = 0.2974 p = 0.0289 ^†^	R = 0.2988 p = 0.0282 ^†^
VEGF/sVEGFR-2 serum	R = 0.3155 p = 0.0256 ^†^	R = 0.2134 p = 0.0002 *	R = 0.2863 p = 0.0003 *	R = 0.2282 p = 0.0047 *

^†^—significant value before Bonferroni correction; *—significant value after Bonferroni correction. Abbreviations: SSc—systemic sclerosis; MMP-9—matrix metalloproteinase-9; TIMP-1—tissue inhibitor of metalloproteinases-1; TIMP-2—tissue inhibitor of metalloproteinases-2, VEGF—vascular endothelial growth factor; sVEGFR-2—soluble form of vascular endothelial growth factor receptor 2.

## Supplementary Materials

The following are available online at https://www.mdpi.com/1422-0067/21/22/8703/s1, Figure S1: Title. Clinical and radiological pictures of Patients 1 and 2 hands with characteristic appearance of sclerodactylia. Table S1: Title. Calculation of TIMP and MMP molecular weights for stoichiometric analysis.

## Abbreviations

ACR	American Rheumatism Association
DED	dry eye disease
dSSc	diffuse systemic sclerosis
ECM	extracellular matrix
ELISA	enzyme-linked immunosorbent assay
EULAR	European League Against Rheumatism
FA	fluorescein angiography
lSSc	limited systemic sclerosis
MMPs	matrix metalloproteinases
MMP-9	matrix metalloproteinase-9
MMP-2	matrix metalloproteinase-2
mRss	modified Rodnan skin score
NVC	nailfold videocapillaroscopy
OCT	optical coherence tomography
SD	standard deviation
SSc	systemic sclerosis
sVEGFR-2	soluble VEGF receptor-2
TFBUT	tear film break-up time
TIMPs	tissue inhibitors of metalloproteinases
TIMP-1	tissue inhibitor of metalloproteinases-1
TIMP-2	tissue inhibitor of metalloproteinases-2
VEGF	vascular endothelial growth factor
