# Supplementary material for: Matrix Metalloproteinases MMP-2 and MMP-9, Their Inhibitors TIMP-1 and TIMP-2, Vascular Endothelial Growth Factor and sVEGFR-2 as Predictive Markers of Ischemic Retinopathy in Patients with Systemic Sclerosis—Case Series Report
Source: Int J Mol Sci. 2020 Nov 18;21(22):8703. doi: 10.3390/ijms21228703 (PMC7698901; doi:10.3390/ijms21228703)
Supplement: Supplementary file 1 [file ijms-21-08703-s001.pdf]

Supplementary Table S1. Calculation of TIMP and MMP molecular weights for stoichiometric analysis.

Protein	UniProtKB ID	AAs	MW (Da)
TIMP1	P01033	24-207	20 708.8
TIMP2	P16035	27-220	21755.0
MMP1	P03956	20-469	51844.2
MMP2	P08253	30-660	70975.7
MMP9	P14780	20-707	76370.5
VEGFA	P15692	27-232	23894.62
sVEGFR-2	P35968	20-1356	20708.83

Molecular weight (MW) given in Daltons (Da) was calculated using the amino acid sequence (AAs) stated (signal peptide sequence removed), retrieved from the UniProt database.

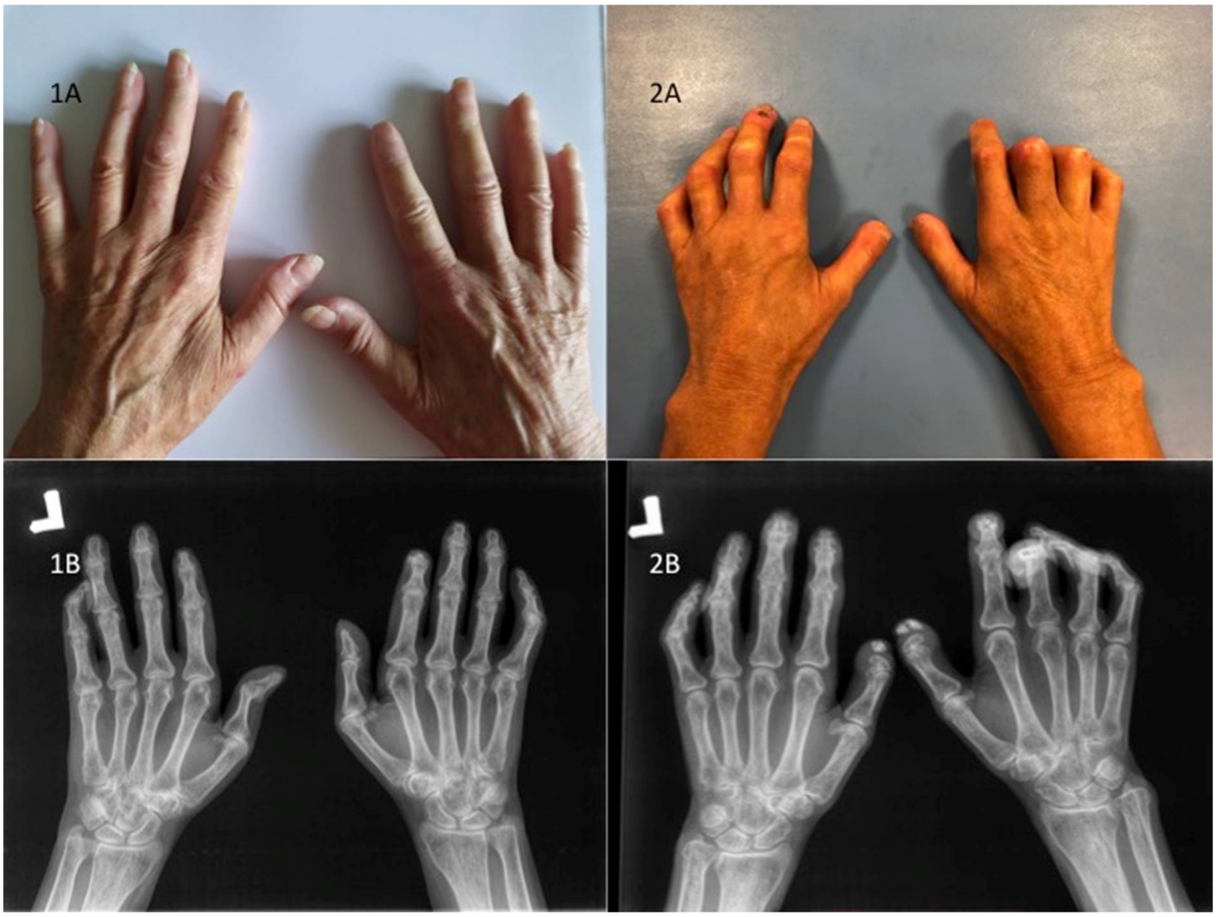

Supplementary Figure S1. Clinical and radiological pictures of Patients 1 and 2 hands with characteristic appearance of sclerodactylia.

Patient 1. In the orthopeadic examination we have found pathological changes in the both hands manifested with thickening of the skin and subcutaneous tissue. The palpable fibrosis of the palmar apponeurosis and flexores muscles tendons of the fingers as well as moderate swelling of proximal

interphalangeal joints (PIP) of 2nd-4th fingers of both hands were also seen (**1A**). The physical examination also revealed limited extension in proximal and distal interphalangeal joints (PIP, DIP) of 3rd-5th fingers of the right hand, limited extension in distal interphalangeal joints (DIP) of 3rd-4th fingers of the left hand and slight flexion in metacarpophalangeal joints (MCP) (**1A**). The limited abduction of the thumbs of both hands was also seen. The digital ulcers (DU) of fingertips of the 2nd and 3th finger of the right hand were also seen (**1A**). The x-ray examination of the hands revealed resorption of 2nd distal phalanx of right hand, joint space narrowing of MCP, PIP and DIP joints of the fingers, errosions and juxta-articular osteopenia (**1B**)

Patient 2. In the orthopeadic examination we have found pathological changes in the both hands manifested with thickening of the skin and subcutaneous tissue. The palpable fibrosis of the palmar apponeurosis and flexoris muscles tendons of the fingers were also seen. These changes resluted in limited extension in proximal and distal interphalangeal joints (PIP, DIP) and flexion in metacarpophalangeal joints (MCP), which led to claw-type deformity of the fingers, specialy of 3rd, 4th and 5th finger of the right hand and 4th and 5th finger of the left hand (**2A**). The digital ulcers (DU) of the 3th finger of the left hand were also seen (**2A**). The x-ray examination of the hands revealed resorption of bilateral 1st, 2nd and 3rd distal phalanxes, joint space narrowing of MCP, PIP and DIP joints of the fingers, errosions, juxta-articular osteopenia, acro-osteolysis of the distal phalanxes of the thumbs and calcinosis of the first fingertips (**2B**).
